# A Tutorial on Mechanical Sensors in the 70th Anniversary of the Piezoresistive Effect

**DOI:** 10.3390/s24113690

**Published:** 2024-06-06

**Authors:** Ferran Reverter

**Affiliations:** Department of Electronic Engineering, Universitat Politècnica de Catalunya–BarcelonaTech, Castelldefels, 08860 Barcelona, Spain; ferran.reverter@upc.edu; Tel.: +34-934137076

**Keywords:** capacitive sensor, mechanical sensor, MEMS, piezoelectric effect, piezoelectric sensor, piezoresistive effect, piezoresistor, strain gauge

## Abstract

An outstanding event related to the understanding of the physics of mechanical sensors occurred and was announced in 1954, exactly seventy years ago. This event was the discovery of the piezoresistive effect, which led to the development of semiconductor strain gauges with a sensitivity much higher than that obtained before in conventional metallic strain gauges. In turn, this motivated the subsequent development of the earliest micromachined silicon devices and the corresponding MEMS devices. The science and technology related to sensors has experienced noteworthy advances in the last decades, but the piezoresistive effect is still the main physical phenomenon behind many mechanical sensors, both commercial and in research models. On this 70th anniversary, this tutorial aims to explain the operating principle, subtypes, input–output characteristics, and limitations of the three main types of mechanical sensor: strain gauges, capacitive sensors, and piezoelectric sensors. These three sensor technologies are also compared with each other, highlighting the main advantages and disadvantages of each one.

## 1. Introduction

In the era of *information and communication technology*, technological ecosystems such as *wireless sensor networks* and the *Internet of Things* (IoT) [1] are widely deployed in our society. Thanks to these advancements, more data/information is available about the status of many *things*, such as our cars, buildings, and cities. This information is processed to improve safety, efficiency, sustainability, mobility, etc., and hence, people’s quality of life. However, in order to acquire such information, it is required, in the first place, to have a set of sensors for the measurement of, for instance, the tire pressure in a smart car, the vibration level in a smart building, or the carbon monoxide concentration in air in a smart city.

**Figure 1:**
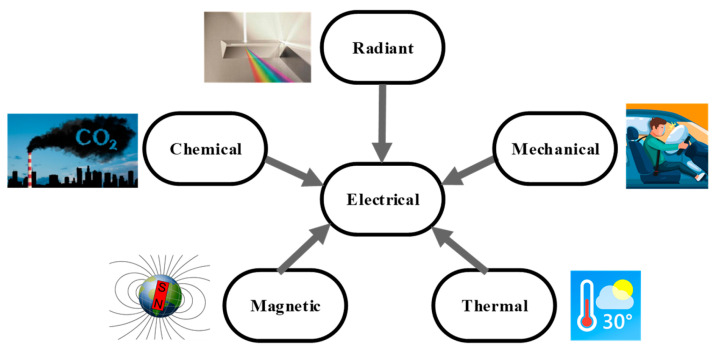
A sensor acquiring information from different energy domains and converting it to the electrical domain.

Sensors are the first (and, probably, the most important) block in the measurement chain of an electronic instrumentation system. Many definitions of a sensor can be found in the literature, but, from [2], a sensor is a device that converts information from a given energy domain to the electrical domain. In other words, when a sensor is subjected to a thermal, mechanical, radiant/optical, chemical, or magnetic signal, its output shows an electrical signal that changes according to the non-electrical signal applied to the input, as graphically represented in Figure 1. The electrical signal at the output of the sensor is generally an analog signal in the form of a resistance, capacitance, inductance, voltage, current, or charge. This signal is then processed in the analog domain by a signal conditioning circuit and, afterwards, converted to digital using an analog-to-digital converter.

This work focuses on mechanical sensors, i.e., sensors that convert information from the mechanical to the electrical domain, usually with an output signal in the form of a resistance, capacitance, or charge. Several mechanical measurands (such as pressure, acceleration, inclination, vibration, weight, deformation, deterioration, displacement, and position) are of interest for industrial, automotive, aerospace, medicine, consumer electronics, home appliances, and research applications. For example, a brand-new car nowadays incorporates at least a hundred sensors [2] and most of them belong to the mechanical group. The widespread incorporation of airbag acceleration sensors around twenty years ago is considered to be a key element in improving safety in cars; this was the beginning of the concept of *smart car*. Another example: the structural fatigue testing of the Airbus A350 aircraft requires more than 12,000 sensors that monitor the structural integrity of the wings when these are subjected to a bending force that generates a vertical displacement of several meters.

The field of sensors, but specifically that related to mechanical sensors, has been highly and positively impacted by the introduction of micro- and nano-electro-mechanical systems [3] (MEMS and NEMS, respectively) technologies. A historic remark: the term MEMS was first introduced by Professors Jacobsen and Wood from the University of Utah in 1986 in the course of writing a proposal to the Defense Advanced Research Projects Agency. Thanks to these technologies, it is possible to embed mechanical structures at a microscopic scale (such as a membrane in a pressure sensor or a seismic mass in an acceleration sensor) together with the electronics (i.e., sensors and the corresponding signal conditioners) into the same integrated circuit. The use of MEMS offers three main advantages: (1) they are small and lightweight, making them suitable for portable and miniaturized applications; (2) they typically require low power, making them suitable for battery-powered applications; and (3) they can be mass-produced using semiconductor fabrication techniques, leading to cost savings. However, MEMS devices are not exempt from limitations; for instance, they are quite sensitive to mechanical shock and vibration, which can limit their reliability in harsh environments.

**Figure 2:**
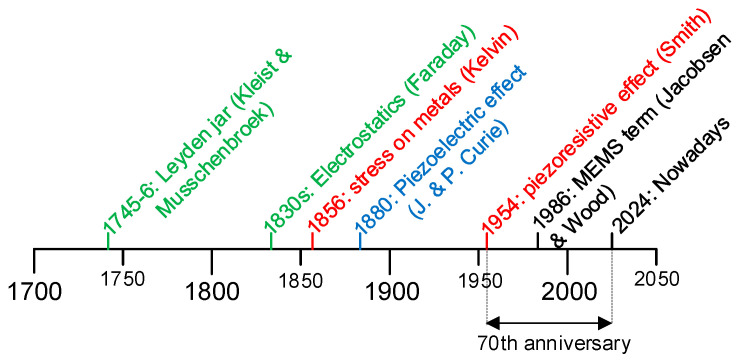
Historic scientific events related to strain gauges (in red), capacitive sensors (in green), and piezoelectric sensors (in blue).

Exactly seventy years ago, in 1954, a highly remarkable event related to the physics of mechanical sensors was announced for the first time [4], as graphically represented in Figure 2 together with other historic scientific events related to mechanical sensors [5,6,7]. Note that many such scientific events occurred in the 19th century, similar to the history of the physics related to thermal sensors, such as the Seebeck effect [8]. The event announced in 1954 was the discovery of the *piezoresistive effect* (originally referred to as the *piezoresistance effect*), which was reported by C. S. Smith while he was visiting the Bell Telephone Laboratories in New Jersey. It is worth noting that the word “piezo” has a Greek origin and means to squeeze or press. According to the piezoresistive effect, the resistivity of a doped semiconductor depends on the applied mechanical stress. Thanks to this, it was possible to design semiconductor strain gauges with a sensitivity much higher than that obtained before in conventional metallic strain gauges. In turn, this motivated the later development of the earliest micromachined silicon devices and the corresponding MEMS and NEMS devices. 

To commemorate this 70th anniversary of the discovery of the piezoresistive effect, a tutorial on mechanical sensors is presented herein. The three main types of mechanical sensor (strain gauges, capacitive sensors, and piezoelectric sensors) are described and compared with each other. For each sensor technology, there is an explanation of the operating principle, subtypes, input–output (I/O) characteristics, and limitations. Section 2 focuses on strain gauges, Section 3 on capacitive sensors, Section 4 on piezoelectric sensors, and finally Section 5 provides a comparison between them.

## 2. Strain Gauges

The first type of mechanical sensor explained here is the strain gauge, which offers a resistance at the output that changes with the mechanical quantity being sensed. The term “gauge” has a French origin (in modern French, the corresponding term is “jauge”) and it means “instrument for measuring”. Accordingly, a strain gauge is a device for measuring the mechanical tension (or strain) affecting a mechanical structure.

### 2.1. Principle

**Figure 3:**
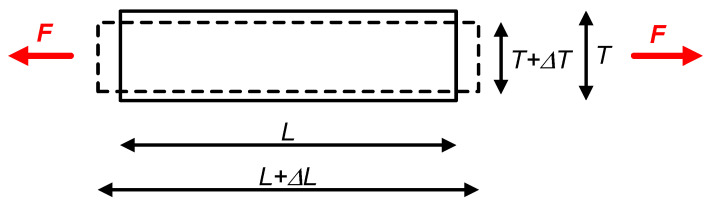
Bar exposed to an external force generating a longitudinal and a transverse strain.

Let us consider a longitudinal structure as a bar of a certain material (with a length *L*, a sectional area *A*, and a diameter *T*) exposed to an external force *F* in the same longitudinal direction, as shown in Figure 3. In such conditions, this bar is subjected to a mechanical stress (σ) that can be calculated as *F*/*A*. Depending on the direction of the external force, the bar suffers from either elongation (with an increase in *L*) or contraction (with a decrease in *L*). The relative change in length (i.e., Δ*L*/*L*) is called the strain (ε) and is usually expressed in µm/m or mm/m. It is assumed that ε > 0 corresponds to elongation, whereas ε < 0 corresponds to contraction. In the elastic region of the material, there is a linear relationship between σ and ε. This relation is defined by Hooke’s Law: ε = σ/*E*, where *E* is the Young’s modulus of the material (for example, *E* = 206 GPa for iron). Note, however, that the bar in Figure 3 is not only subjected to a longitudinal deformation, but also to a transverse deformation. In other words, when the bar is exposed to elongation, it becomes longer but also thinner (as represented in Figure 3), whereas when the bar is exposed to contraction, it becomes shorter but also thicker. Consequently, we have a longitudinal strain (ε_L_ = Δ*L*/*L*) and a transverse strain (ε_T_ = Δ*T*/*T*), and these have opposite signs, i.e., if Δ*L* > 0 then Δ*T* < 0 and vice versa. The absolute value of the ratio between these two strains is known as the Poisson’s ratio (i.e., υ = |ε_T_/ε_L_|) that depends on the bar material, but it generally takes a value around 0.3 for most metals.

After explaining the basics of materials engineering, let us proceed with the definition of a strain gauge. First of all, a strain gauge belongs to the category of resistive sensors and, hence, it offers an electrical resistance (*R*) that can be expressed as [9]:

(1) $$R=\rho \frac{L_{s}}{S},$$

where *ρ* is the resistivity, *L*_s_ is the length, and *S* is the cross-sectional area of the sensor material. The basic principle behind a strain gauge is that its resistance depends on the applied mechanical stress. This principle affecting metallic materials was first reported by William Thomson (Lord Kelvin) in 1856 [6], whereas that affecting semiconductor materials was reported by C. S. Smith in 1954 [4], exactly 70 years ago. Using as a reference the bar shown in Figure 3, the gauge is pasted on the mechanical structure so as to suffer from the same strain. In other words, the effective length of the sensor changes from *L*_s_ to *L*_s_ + Δ*L*_s_ due to the mechanical stress, but the ratio Δ*L*_s_/*L*_s_ is equal to Δ*L*/*L* affecting the bar, provided that the gauge is correctly installed. In order to quantify the change in resistance due to the strain, a strain gauge is specified with the corresponding gauge factor (*K*) that is defined as [9]:

(2) $$K=\frac{\Delta R/R}{\Delta L_{s}/L_{s}}$$

As explained in more detail in the next subsection, the factor *K* highly depends on the material employed for the manufacturing of the gauge.

### 2.2. Types

Depending on the material employed for manufacture, strain gauges can be classified in two subgroups: metallic and semiconductor. 

**Figure 4:**
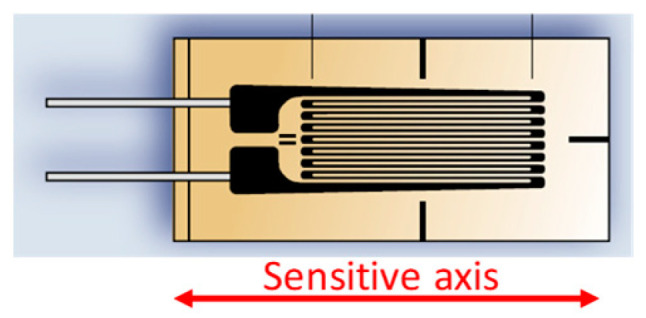
Typical commercial metallic strain gauge with a serpentine shape.

Metallic strain gauges are made of a thin film of metal (e.g., copper–nickel alloy, also known as constantan) in a serpentine shape, as shown in Figure 4, placed on a thin film of a plastic base (e.g., polyimide). The thickness of the metal is in units of microns, whereas that of the plastic is in tens of microns, but the length of the gauge is in the millimeter range. The change in resistance in a metallic strain gauge is mostly due to the geometrical deformation of the metal, i.e., both *L*_s_ and *S* in (1) change in the event of a mechanical stress, as initially reported by Lord Kelvin in 1856 [6]. Assuming the sensitive axis shown in Figure 4, when the gauge is under elongation, *L*_s_ increases but *S* decreases, thus increasing *R* in (1). On the other hand, when the gauge is under contraction, *L*_s_ decreases but *S* increases, thus decreasing *R*. The resulting *K* differs depending on the metallic material employed, but it is around 2 for copper–nickel alloys and 2.2 for chromium–nickel alloys.

Semiconductor strain gauges (also so-called piezoresistors) are made of a doped semiconductor material (e.g., p-type silicon). These gauges rely on the *piezoresistive effect*, which was initially announced by C. S. Smith in 1954 [4]. According to this effect, when a doped semiconductor material is under mechanical stress, it undergoes a significant change in its resistivity and, hence, of its resistance. Therefore, unlike what occurs in metallic strain gauges, the mechanical stress induces mostly resistivity changes (i.e., *ρ* in (1)) in semiconductor strain gauges. The resulting factor *K* is generally much higher. This can easily be around 100, thus resulting in a sensitivity that is 50 times higher than that obtained in a metallic gauge. N- and p-type piezoresistors exhibit opposite trends in resistance change under stress [9].

**Figure 5:**
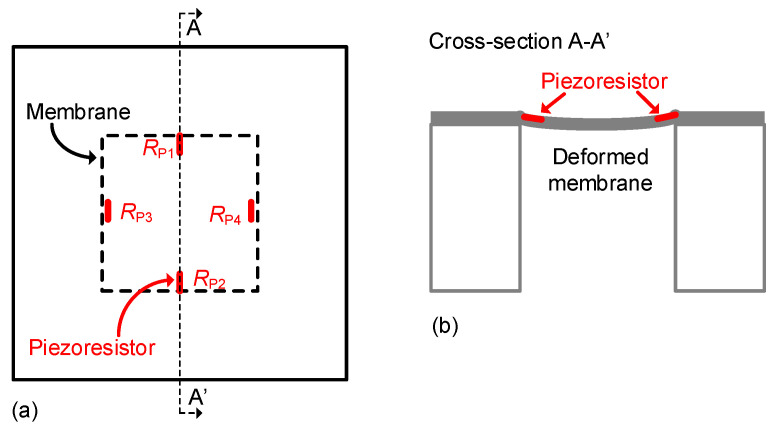
Piezoresistive pressure sensor based on a membrane including four piezoresistors. (**a**) Top view. (**b**) Cross-section A-A’.

**Figure 6:**
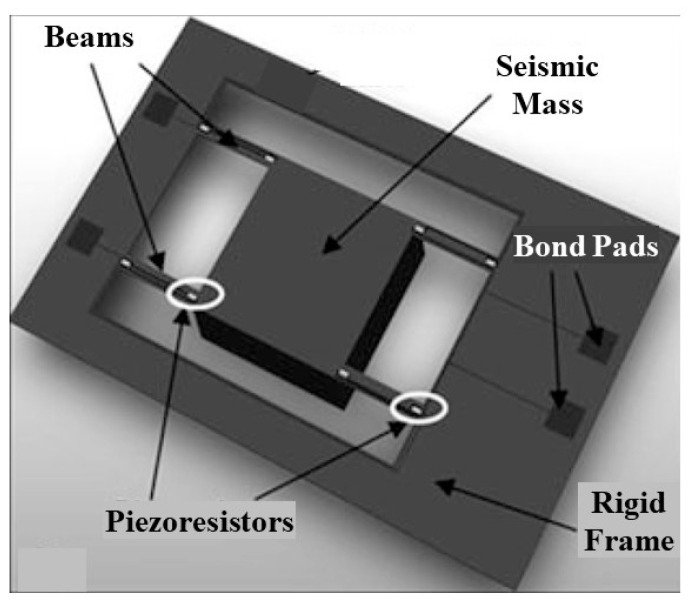
Piezoresistive acceleration sensor based on a flexure beam–seismic mass structure.

Metallic strain gauges are generally implemented at a macroscopic scale, although these can also be integrated into microstructures, such as a membrane [10]. In contrast, semiconductor strain gauges are usually realized at a microscopic scale, although these are also commercially available with dimensions similar to those in Figure 4 but replacing the serpentine of metal by a bar (of a few millimeters) of a doped semiconductor. Macroscopic strain gauges are pasted on the mechanical structure under test, whereas microscopic ones are strategically embedded into a MEMS device. For example, in a piezoresistive pressure sensor based on a membrane, the piezoresistors are located near the edge of the membrane, which is where the maximum stress occurs, as represented in Figure 5 [9]. In a piezoresistive accelerometer based on a flexure beam–seismic mass structure attached to a rigid frame, the piezoresistors are located at the stress-maximum positions of the beam (i.e., at the root of the flexure), as represented in Figure 6 [11]. Such integrated piezoresistors are formed using an impurity-doping technique, such as diffusion, ion implantation, or epitaxy [9].

### 2.3. I/O Characteristic

A generic relation between the applied strain (ε) and the output resistance (*R*) of a strain gauge can be expressed, in a linear approximation, as [12]:

(3) $$R=R_{0}\left(1+K\cdot \varepsilon \right),$$

where *R*_0_ is the gauge resistance at rest that generally ranges from 100 and 1000 Ω. According to the commercial devices available on the market, the maximum ε is around 50,000 μm/m (5%) for metallic strain gauges, but it is lower (a factor of 10 [13]) for semiconductor ones. Additionally, the change in resistance with strain is less linear in semiconductor gauges [13]. Considering a typical metallic strain gauge with *R*_0_ = 120 Ω, *K* = 2, and ε_max_ = 5%, the gauge resistance changes from 120 to 132 Ω at full scale.

**Figure 7:**
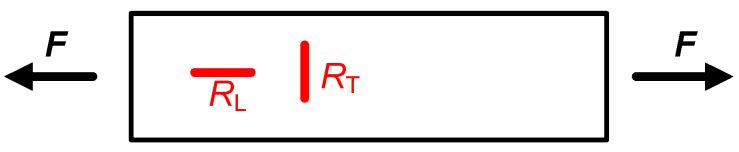
Bar subjected to a longitudinal force including two strain gauges: *R*_L_ in a longitudinal direction, and *R*_T_ in a transverse direction.

Let us assume the case of a longitudinal structure (such as a bar) subjected to a longitudinal force, as represented in Figure 7, with a homogenous deformation along the bar. If the gauge is placed in the longitudinal direction (i.e., *R*_L_ in Figure 7), this can be expressed as [12]:

(4) $$R_{L}=R_{0}\left(1+K\cdot \varepsilon_{L}\right)$$

However, if the gauge is placed in the transverse direction (i.e., *R*_T_ in Figure 7), the corresponding relationship is [12]:

(5) $$R_{T}=R_{0}\left(1+K\cdot \varepsilon_{T}\right)=R_{0}\left(1-K\cdot \upsilon \cdot \varepsilon_{L}\right)$$

In the scenario shown in Figure 7, it is quite typical to have a topology with four strain gauges (two longitudinal and two transverse) interconnected in a Wheatstone bridge so as to increase the sensitivity of the output signal and also to compensate for resistance changes due to temperature [9]. Actually, there are commercial devices that already include the four gauges in such positions and interconnected in a bridge.

**Figure 8:**
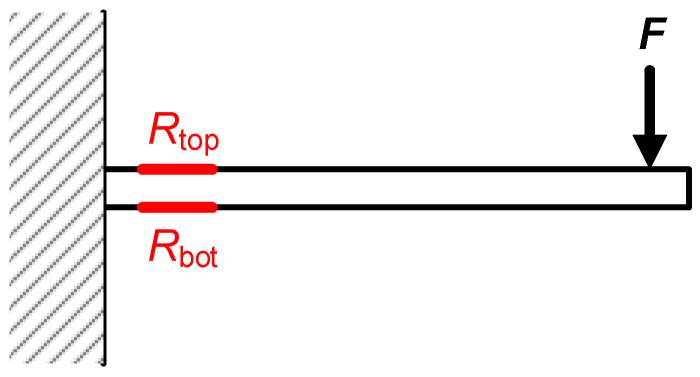
Cantilever beam subjected to a bending force including two strain gauges, one at the top and the other at the bottom at the root of the flexure.

Another typical scenario of application of strain gauges is a cantilever beam subjected to a bending force, as shown in Figure 8. In such a case, the maximum stress occurs at the fixed end of the cantilever [14] (i.e., at the root of the flexure), and this is the most appropriate place to locate the strain gauges. Here, it is quite typical to employ at least two strain gauges (one at the top (*R*_top_) and another at the bottom (*R*_bot_), as illustrated in Figure 8) with opposite variations and interconnected in a Wheatstone bridge so as to increase the sensitivity of the measurement. Accordingly, the I/O characteristic of the gauges in Figure 8 can be expressed as [12]:

(6) $$R_{top}=R_{0}\left(1+K\cdot \varepsilon_{top}\right)$$

(7) $$R_{bot}=R_{0}\left(1+K\cdot \varepsilon_{bot}\right)=R_{0}\left(1-K\cdot \varepsilon_{top}\right),$$

where ε_top_ and ε_bot_ are the strains affecting, respectively, the top and bottom of the cantilever at the root of the flexure, which are equal in magnitude but with opposite signs (i.e., ε_bot_ = −ε_top_).

For piezoresistors embedded into MEMS devices (such as in Figure 5 and Figure 6), it is quite common to model the I/O characteristic through the piezoresistive coefficients instead of the gauge factor. Accordingly, the relative change in resistance due to the mechanical stress can be modeled as [14]:

(8) $$\frac{\Delta R}{R_{0}}=\pi_{L}\cdot \sigma_{L}+\pi_{T}\cdot \sigma_{T},$$

where π_L_ and π_T_ are the longitudinal and transverse piezoresistive coefficients, whereas σ_L_ and σ_T_ are the longitudinal and transverse stresses affecting the gauge, respectively. 

For the pressure sensor shown in Figure 5, the membrane suffers from a deflection when it is exposed to a pressure difference between its two faces. Such a deflection causes the same stress (say, σ) to the four piezoresistors, but *R*_P1_ and *R*_P2_ are affected longitudinally, whereas *R*_P3_ and *R*_P4_ are affected transversally. In addition, for p-type silicon piezoresistors, we have π_L_ ≈ −π_T_ [14]. In such conditions, applying (8), the piezoresistors in Figure 5 can be expressed as:

(9) $$R_{P1}=R_{P2}=R_{0}\left(1+\pi_{L}\cdot \sigma \right)$$

(10) $$R_{P3}=R_{P4}=R_{0}\left(1-\pi_{L}\cdot \sigma \right)$$

Therefore, similar to the cases explained before, two resistances increase and the other two decrease but by the same magnitude. Again, these four piezoresistors in Figure 5 are then usually interconnected in a bridge topology to increase the overall sensitivity.

### 2.4. Limitations

The main limitation of strain gauges is the occurrence of thermal drifts, and these are more significant for the semiconductor types than for the metallic ones. This is because the resistance of silicon and the piezoresistive effect highly depend on the temperature [9]. In order to compensate for these thermal effects, some mechanical sensors include a thermal sensor [8], thus resulting in a thermally compensated mechanical sensor. For example, in a mechanical sensor with a Wheatstone bridge topology, the thermal sensor generates an increase in the supply voltage of the bridge to compensate for the decrease in the bridge output voltage due to an increase in temperature. Another alternative to compensate for the thermal effects is the inclusion of a passive strain gauge [12]. In such a case, the measurement system, on the one hand, has an active strain gauge that is subjected to both mechanical stress and thermal changes, and, on the other hand, a passive strain gauge only affected by thermal changes. These two gauges (active and passive) are then appropriately interconnected in a Wheatstone bridge. Also related to the thermal effects, it is important to take care of the self-heating of the strain gauge. In that sense, the excitation current of the gauge should be of a few units of milliamperes or even lower.

Another limitation in the measurement of strain gauges is the effect of the parasitic resistances (which also depend on temperature) of the interconnecting cable, especially when the gauge resistance is low (say, 100 Ω or lower) and the cable is long. In order to avoid such a limitation, the strain gauge can be measured by applying the 4-wire technique, as also suggested for resistance temperature detectors, such as Pt100 [8]. When this technique is applied, a couple of cables are used to inject the current, and another couple for measuring the voltage drop exclusively between the terminals of the strain gauge [13]. An alternative is the three-wire technique [15], which is less accurate but requires only three interconnecting cables to the sensor.

## 3. Capacitive Sensors

The second kind of mechanical sensor discussed herein is the capacitive sensor, which provides a capacitance at the output that changes with the measurand. Although capacitive sensors can also be employed to measure non-mechanical signals (such as relative humidity [16] or gas concentration), this section is focused on mechanical capacitive sensors.

### 3.1. Principle

The beginning of capacitor technology is generally attributed to Ewald Georg von Kleist in 1745 and Pieter van Musschenbroek in 1746 [5], who developed a capacitor that is known as the Leyden jar. However, the major contributions to the field of electrostatics and capacitances were made by Michael Faraday in the decade of 1830s. Among others, he (i) discovered that the charge stored in a capacitor is directly proportional to its capacitance and the applied voltage, (ii) introduced the concept of the dielectric constant, (iii) invented the first practical fixed and variable capacitors, and iv) introduced the concept of Faraday’s Cage. The contributions of Faraday to the capacitor technology were so important that they were recognized by using his name in the unit for capacitance (Farad) in the international system of units. It is worth highlighting that Faraday is also considered to be the creator in 1833 of the first thermistor, which is one the main thermal sensor technologies [8]. In addition, Faraday was the assistant of Sir Humphrey Davy, who announced in 1821 for the first time the physics behind a resistance temperature detector (RTD), which is another of the main thermal sensor technologies [8]. Accordingly, many remarkable events related to sensor technology were announced in a short period of time at the end of the industrial revolution by scientists from the same school. A similar situation is highlighted in Section 4.1.

**Figure 9:**
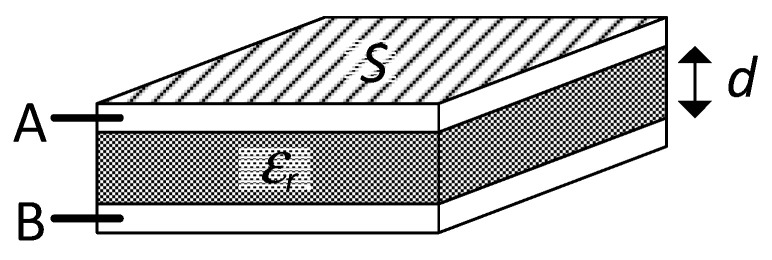
Capacitance with a parallel plate topology.

In order to explain the operating principle of capacitive sensors, let us assume first a capacitance with a parallel plate topology, as shown in Figure 9. This is formed by two face-to-face electrodes or plates (A and B) and an intermediate dielectric material. In such a topology, the capacitance (*C*) between A and B can be expressed, neglecting edge effects, as [17]:

(11) $$C=\varepsilon_{0}\varepsilon_{r}\frac{S}{d},$$

where ε_0_ is the vacuum permittivity (i.e., 8.85 pF/m), ε_r_ is the relative permittivity of the dielectric material, *S* is the overlap area between electrodes, and *d* is the distance between the electrodes. 

**Figure 10:**
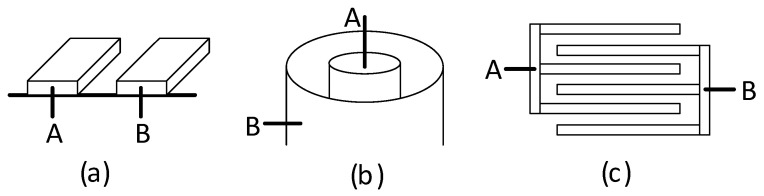
Capacitive sensors with electrodes A and B in (**a**) co-planar, (**b**) cylindrical, and (**c**) interdigital topologies.

According to (11), a capacitive sensor can be defined as a sensing device whose output capacitance changes with the measurand because this alters *d*, *S*, and/or ε_r_, although the effects on *d* are the most common. Of course, capacitive sensors are not limited to the simple electrode topology represented in Figure 9 and can be implemented using other configurations, such as the co-planar, cylindrical, and interdigital topologies shown in Figure 10a–c, respectively. In such cases, the value of the capacitance cannot be determined by (11), although the capacitance still depends on the intermediate dielectric and the geometry (i.e., area and distance). 

### 3.2. Types

Capacitive sensors can be classified in several ways. Some of these classifications are explained next.

In terms of the potential applied to the electrodes, two subtypes of capacitive sensors can be identified [18]: (1) floating capacitive sensors (FCS), in which the two electrodes are not connected by default to any potential and, hence, they are available to the measurement circuit; and (2) grounded capacitive sensors (GCS), also known as one-terminal capacitive sensors [19], in which one of the two electrodes is always connected to ground. Although FCS are more attractive than GCS in terms of circuit design, the use of GCS is mandatory in some scenarios since the ground connection of one of the sensor electrodes is imposed by the application itself. A typical example is the level measurement of a conductive liquid inside a metallic tank that is grounded for safety reasons [20]. In such a case, an isolated metal rod is one of the sensor electrodes, whereas the other is the grounded shell of the tank. Other examples where GCS are required are the distance/proximity measurement to a grounded metallic object [21], and the linear/angular displacement measurement of a grounded shaft [22].

**Figure 11:**
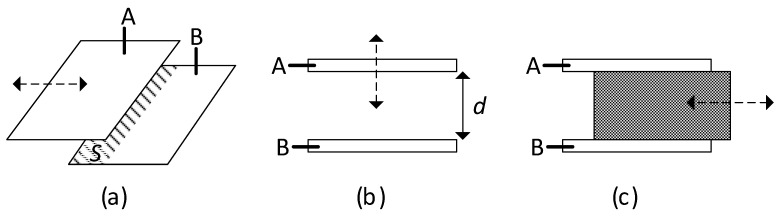
Single-element capacitive sensor where the displacement to be measured causes a variation in (**a**) the overlap area, (**b**) the distance between electrodes, and (**c**) the properties of the intermediate dielectric.

**Figure 12:**
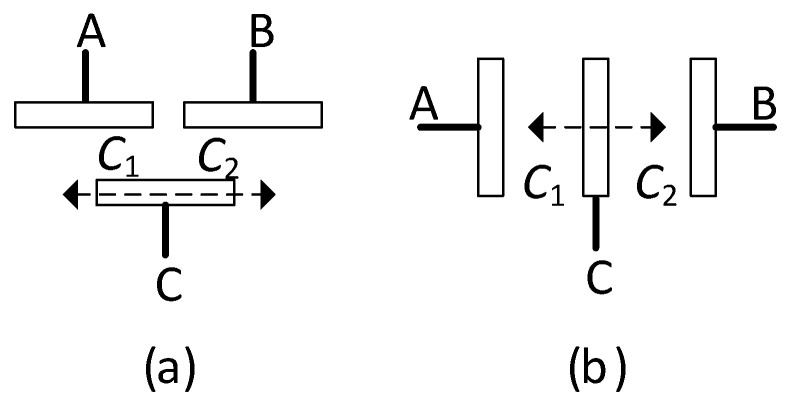
Differential capacitive sensor where the displacement to be measured causes a variation in (**a**) the overlap area, and (**b**) the distance between electrodes.

In terms of the number of sensing elements, capacitive sensors can be classified in two subtypes: single-element and differential [23]. A single-element capacitive sensor (SCS) just requires a couple of electrodes (A and B), thus resulting in a single capacitive sensing element. A displacement changes the overlap area, the distance between electrodes, or the properties of the intermediate dielectric of the SCS, as represented in Figure 11a–c, respectively; cases shown in Figure 11a,b are the most common in mechanical applications. On the other hand, a differential capacitive sensor (DCS) involves three electrodes (A, B, and C, where C is a movable electrode) and two capacitive sensing elements (*C*_1_ between A and C, and *C*_2_ between B and C) that change in opposite directions. As represented in Figure 12, a displacement of electrode C to the left generates an increase in *C*_1_ but a decrease in *C*_2_, whereas a displacement to the right causes opposite variations. In Figure 12a, the displacement of electrode C brings about a change in the overlap area, whereas in Figure 12b, a change in the distance between electrodes. Similar to the case of using several strain gauges with opposite variations explained in Section 2, the use of a DCS has advantages in terms of sensitivity and also in terms of linearity, as highlighted in the next subsection. Finally, note that any mechanical magnitude causing a displacement (such as pressure, force, and acceleration) can be measured using the capacitive sensors shown in Figure 11 and Figure 12.

**Figure 13:**
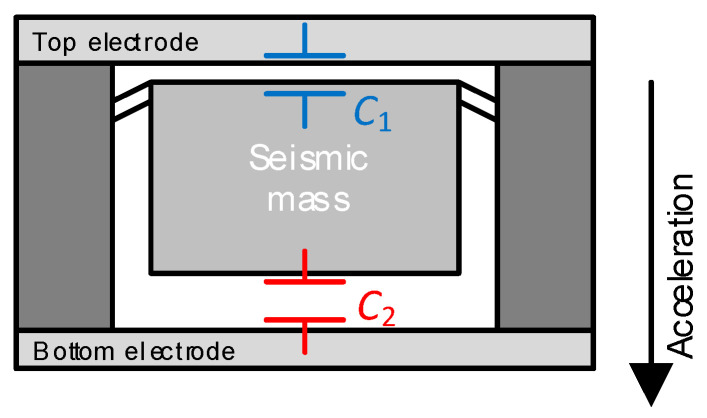
Acceleration sensor based on a capacitive MEMS with a differential topology.

In a similar manner to what was explained in Section 2.2 for strain gauges, capacitive sensors (with a single-ended or a differential topology) can be implemented as either a macrodevice or a microdevice integrated into a MEMS. Macro-capacitive sensors usually offer a capacitance in the range of tens or hundreds of picofarads, whereas micro-capacitive sensors, a few units of picofarads and even lower. For example, the capacitive accelerometer in [24] offers a capacitance of 1.5 pF at rest, and a sensitivity of 0.1 pF/g in the measuring range of ±2 g. Similar to the MEMS topologies shown before in Figure 5 and Figure 6, in a capacitive pressure sensor, a pressure difference generates a deflection of the membrane, whereas in a capacitive accelerometer, an acceleration causes a movement of the seismic mass. In both cases, this is generally translated into a variation in the distance between electrodes and, hence, a change in the sensor capacitance. As an example, Figure 13 shows a capacitive accelerometer based on a DCS. When there is an acceleration in the direction indicated in Figure 13, the seismic mass (which behaves as a movable electrode, i.e., electrode C in Figure 12) moves up and, hence, *C*_1_ increases and *C*_2_ decreases, where *C*_1_ is the capacitance between the top electrode and the seismic mass and *C*_2_ between the bottom electrode and the mass. For two- or three-axis accelerometers, the device can include two or three independent microstructures (i.e., one seismic mass for each axis), or just a single seismic mass for all axes [25]. The former approach allows each structure to be optimized individually and reduces problems related to cross-axis sensitivity, but it requires a larger layout.

### 3.3. I/O Characteristic

Most mechanical capacitive sensors suffer from geometrical variations, i.e., changes in the overlap area (Figure 11a and Figure 12a) or distance (Figure 11b and Figure 12b) with the measurand. The case where the measurand alters the properties of the intermediate dielectric (Figure 11c) is not modeled here since it is quite unusual in mechanical applications.

For an SCS subjected to area variations (Figure 11a), its capacitance can be expressed, assuming the parallel plate topology in Figure 9, as [12]:

(12) $$C=\varepsilon_{0}\varepsilon_{r}\frac{S_{0}+\Delta S}{d},$$

where *S*_0_ is the overlap area at rest (i.e., when the mechanical input equals zero) and Δ*S* is the (positive or negative) variation in the area caused by the mechanical input under measurement. Assuming $C_{0}=\varepsilon_{0}\varepsilon_{r}S_{0}/d$, (12) can be rewritten as:

(13) $$C=C_{0}\left(1+\frac{\Delta S}{S_{0}}\right),$$

which shows that the capacitance changes linearly with the relative variation in the overlap area (i.e., Δ*S*/*S*_0_). On the other hand, for an SCS exposed to distance variations (Figure 11b), the expression of its capacitance is [12]:

(14) $$C=\varepsilon_{0}\varepsilon_{r}\frac{S}{d_{0}-\Delta d},$$

where *d*_0_ is the distance between the electrodes at rest and Δ*d* is the (positive or negative) variation in distance caused by the mechanical input. Considering now $C_{0}=\varepsilon_{0}\varepsilon_{r}S/d_{0}$, (14) becomes:

(15) $$C=\frac{C_{0}}{1-\frac{\Delta d}{d_{0}}}$$

According to (15), the relation between the capacitance and the relative variation in distance (i.e., Δ*d*/*d*_0_) is not linear here but hyperbolic. Since both Δ*S*/*S*_0_ and Δ*d*/*d*_0_ normally change in a linear relation with the mechanical input (from now on, *x*), expressions (13) and (15) can be rewritten, respectively, as [23]:

(16) $$C=C_{0}\left(1+k\cdot x\right)$$

(17) $$C=\frac{C_{0}}{1-k\cdot x},$$

where *k* is a proportionality constant.

For a DCS subjected to area variations (Figure 12a), the capacitances of the two sensing elements can be directly expressed as [23]:

(18) $$C_{1}=C_{0}\left(1+k\cdot x\right)$$

(19) $$C_{2}=C_{0}\left(1-k\cdot x\right),$$

where *C*_1_ increases and *C*_2_ decreases linearly with *x*. On the other hand, if the DCS is exposed to distance variations (Figure 12b), the two sensing capacitances are [23]:

(20) $$C_{1}=\frac{C_{0}}{1-k\cdot x}$$

(21) $$C_{2}=\frac{C_{0}}{1+k\cdot x},$$

where *C*_1_ increases and *C*_2_ decreases with *x*, but not in a linear relation. However, in a DCS, the mechanical information is not in the value of *C*_1_ or *C*_2_, but in the following ratio of capacitances [23]:

(22) $$M=\frac{C_{1}-C_{2}}{C_{1}+C_{2}}$$

Substituting (18) and (19) into (22) provides *M* = *k* · *x*, but the same is obtained when substituting (20) and (21) in (22). Therefore, the ratio *M* linearly changes with *x* for both scenarios. In addition, cross-sensitivity issues equally affecting *C*_1_ and *C*_2_ are compensated for when (22) is applied. These advantages make DCS more attractive than SCS and, for this reason, DCS are more commonly employed.

Finally, it is worth highlighting that capacitive MEMS (e.g., based on the topology shown in Figure 13) can suffer from non-linearity problems due to the electrostatic forces between electrodes, which can be critical at microscopic scale. Such a problem is usually solved by means of a position-feedback mechanism that counterbalances the applied external force [26].

### 3.4. Limitations

First of all, the limitation related to thermal drifts indicated in Section 2.4 for strain gauges is usually not so critical in capacitive sensors. However, mechanical capacitive sensors are subjected to other limitations, such as:

▪ The read-out circuit for a capacitive sensor is generally more complex than that for a resistive sensor. This is because, on the one hand, an alternating excitation of the sensor is required and, on the other hand, it is necessary to detect very small changes in capacitance (for example, units, tenths, and even hundredths of picofarads).
▪ Considering that capacitive sensors are usually in the range of picofarads, the corresponding impedance is high or even very high. Consequently, these sensors can be quite susceptible to interference coming, for instance, from the mains electricity supply. A good consequence of such a high impedance is that capacitive sensors become a low-power sensing solution.
▪ The measurement of the sensor capacitance can be affected by parasitic capacitances related to the tracks of the printed circuit board or to the interconnecting shielded cable in case the sensor is remote; note that a shielded cable is required to avoid the interference effects indicated before. Scenarios where the parasitic capacitance is clearly higher than the sensor capacitance are quite typical, so that special measurement techniques (such as passive or active shielding [27]) must be applied to avoid the effects of the former.

## 4. Piezoelectric Sensors

The last type of mechanical sensor explained in detail is the piezoelectric sensor, which provides an electrical charge at the output that depends on the value of the mechanical signal being sensed. Piezoelectric sensors belong to the category of *self-generating* sensors, since they are able to provide an output signal with information about the measurand without requiring any external power. In addition, these are *reversible transducers* so that they can behave as a sensor or an actuator depending on whether the input is a mechanical or electrical signal, respectively.

### 4.1. Principle

Piezoelectric sensors rely on the *piezoelectric effect*, which was first observed by the brothers Jacques and Pierre Curie in 1880 [7]; it is worth noting that, in 1895, Pierre Curie married Marie Skłodowska-Curie, the pioneering outstanding scientist on radioactivity. According to the piezoelectric effect, certain materials (identified as piezoelectric materials) are able to generate an electrical charge in response to an applied mechanical stress, and this charge is proportional to the stress and changes sign with it. Considering the reversibility of the process, the term “direct piezoelectric effect” is employed when there is a generation of charge in the event of a mechanical stress, whereas the “converse piezoelectric effect” is used when there is a generation of a mechanical signal due to an electrical signal applied to the input [7]. In addition, the direct effect can be employed in both sensing and energy harvesting applications [28]. 

**Figure 14:**
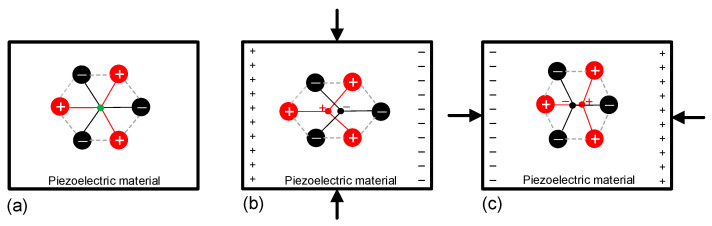
Two-dimensional example of the lattice of a piezoelectric material (**a**) without mechanical stress, (**b**) under a transversal piezoelectric effect, and (**c**) under a longitudinal piezoelectric effect.

The generation of charge is due to a change in the atomic structure of the piezoelectric material when this is under mechanical stress. In a non-piezoelectric material, the centroid (i.e., the geometric center) of the positive charges in a unit cell geometrically coincides (with and without mechanical stress) with that of the negative charges and, hence, these two cancel out and no polarization appears. The situation is not the same, however, in a piezoelectric material since this has a unique distribution of charges. In a first approximation, the unit cell of a piezoelectric material has a hexagonal configuration [29], as graphically represented in Figure 14, where the positive charges correspond to silicon ions and the negative charges to oxygen ions, considering quartz as a piezoelectric material. Without any mechanical stress applied (Figure 14a), the centroids of the positive and negative charges coincide; this is marked by a green dot in Figure 14a. But, under the compression shown in Figure 14b, the material is expanded horizontally and, hence, the positive (negative) charge at the left (right) of the unit cell is moved apart. As a consequence of this, the centroids of the positive and negative charges do not coincide, but they move to the positions indicated by the red and black dots in Figure 14b, respectively, thus generating a microscopic electrical dipole. Considering that this phenomenon occurs to all the unit cells of the material, a macroscopic electrical dipole appears as a combination of all the microscopic dipoles. The result is an accumulation of positive charges on the left face of the material, and an accumulation of negative charges on the right face, as shown in Figure 14b. This is known as the polarization of the electrodes that are placed face-to-face, as in a capacitor. Note that, in the case of Figure 14b, the direction of the electric polarization is perpendicular to that of the mechanical stress; this is known as a *transversal piezoelectric effect* [29]. Figure 14c shows another potential scenario with a *longitudinal piezoelectric effect*, where the direction of the resulting polarization (here with opposite sign with respect to Figure 14b) is in parallel to that of the mechanical stress. Then, if the piezoelectric material in Figure 14b or Figure 14c is placed in a closed circuit, there is a movement of charge from one electrode (face) to the other and, hence, a current can be recorded.

**Figure 15:**
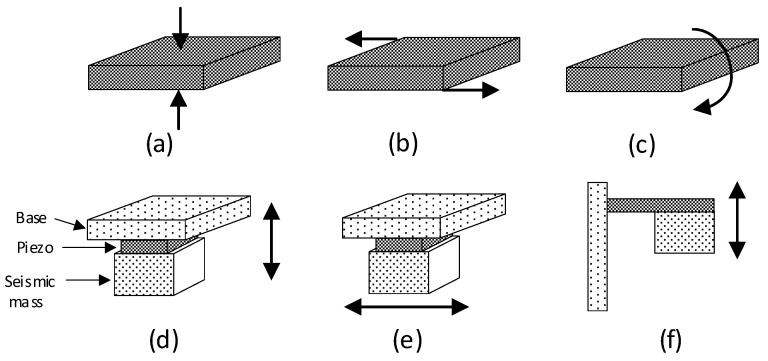
Piezoelectric material subjected to a (**a**) compression, (**b**) shear, and (**c**) bending force. Topologies for the acceleration measurement using a (**d**) compression, (**e**) shear, and (**f**) bending force; the arrow indicates the sensitive axes of the accelerometer.

The generation of charge in a piezoelectric material can be due to different types of applied force, but the most typical are compression, shear, and bending, as shown in Figure 15a–c, respectively. For a piezoelectric sensor intended to measure acceleration or vibrations, one of the faces of the piezoelectric material is attached to a base, whereas the other is attached to a seismic mass, as represented in Figure 15d–f for the compression, shear, and bending cases, respectively. An acceleration in the direction indicated by the arrow in Figure 15d–f causes a movement of the seismic mass that mechanically deforms the piezoelectric material and, hence, an electrical charge is obtained at the output. The case represented in Figure 15e, with a shear stress, is the most typical in commercial piezoelectric sensors.

### 4.2. Types

As occurs with other types of sensors, piezoelectric sensors can be classified in various ways. A first classification is according to the piezoelectric material employed. Piezoelectric materials can be either natural or synthetic. In the natural subgroup, the most common material is quartz. In the synthetic subgroup, many different options are available, but the most popular are lead zirconate titanate (abbreviated as PZT), polyvinylidene fluoride (abbreviated as PVDF), zinc oxide, and aluminum nitride; PZT is a piezoceramic, PVDF is a polymer, whereas the last two are piezoelectric semiconductors. Natural piezoelectric materials generally suffer from fewer thermal drifts, but synthetic piezoelectric materials have a higher sensitivity (at least a factor of 10 higher) and are easier to be mechanized. For instance, sensors based on PVDF can be fabricated in a flexible thin film that can be easily adapted to the application. Considering the growing environmental concern regarding toxicity in lead-containing devices, there are also initiatives to develop new lead-free piezoelectric materials [7]. It is worth noting that the piezoelectric effect has also been reported in biological materials, such as bones [30].

**Figure 16:**
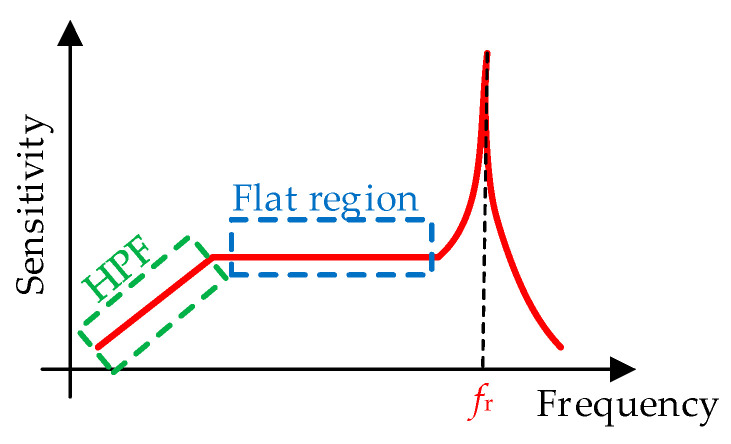
Typical frequency response of a piezoelectric sensor.

A second classification of piezoelectric sensors is according to the operating region in their frequency response. Figure 16 shows the typical frequency response of a piezoelectric sensor, where the frequency is that corresponding to the mechanical input being measured. From Figure 16, there is a high-pass filter (HPF) region at low frequencies that is generally avoided, a flat region at intermediate frequencies, and then a remarkable resonance peak at a frequency *f*_r_. Considering such a frequency response, two subtypes of piezoelectric sensors can be distinguished:

(1) Sensors operating at the flat region, with a sensitivity that is independent of the frequency of the mechanical input. Most mechanical sensors intended to measure force, pressure, and acceleration operate in that region. It is usually recommended to have a maximum measuring frequency five times lower than *f*_r_ so that the sensitivity error is lower than 5%. Reducing the value of the seismic mass in Figure 15 increases the value of *f*_r_ and, hence, the flat region becomes wider. However, this is at the expense of a lower sensitivity. Therefore, there is a bandwidth−sensitivity trade-off.
(2) Sensors operating at the resonance peak, with a very high value of sensitivity. This is the case, for example, of ultrasound sensors intended to measure distance or presence applying the pulse–echo technique. In such a case, the operating frequency is known (e.g., 40 kHz) and coincides with *f*_r_ in Figure 16 so as to achieve the maximum sensitivity. On the other hand, some chemical piezoelectric sensors rely on the fact that a variation in the mass of the chemical substance to be measured changes the resonance frequency of the sensor.

Similar to what is described in Section 2.2 and Section 3.2, piezoelectric sensors can also be implemented as either a macrodevice or a microdevice integrated in a MEMS; in the latter case, the term piezoMEMS is often employed. Most piezoMEMS reproduce the operating principle shown in Figure 15d–f at microscopic scale. For the manufacturing of piezoMEMS, many piezoelectric materials have been tested, but it is quite typical to use a synthetic material, such as PZT, zinc oxide, or aluminum nitride.

### 4.3. I/O Characteristic

As illustrated before in Figure 14, the polarization obtained in a piezoelectric material depends on the direction of the mechanical stress applied. The theory behind piezoelectricity states that the polarization is a first-rank tensor, the stress is a second-rank tensor, whereas the description of the direct piezoelectric effect requires a third-rank tensor [31]. However, thanks to the symmetry of the stress tensor, this can be reduced to a first-rank tensor, to be precise, a 6-dimensional vector when the Voigt notation is applied. Accordingly, the third-rank tensor related to the piezoelectric effect can be reduced to a second-rank tensor, i.e., a 3 × 6 array [31]. These assumptions are considered in the following paragraphs.

**Figure 17:**
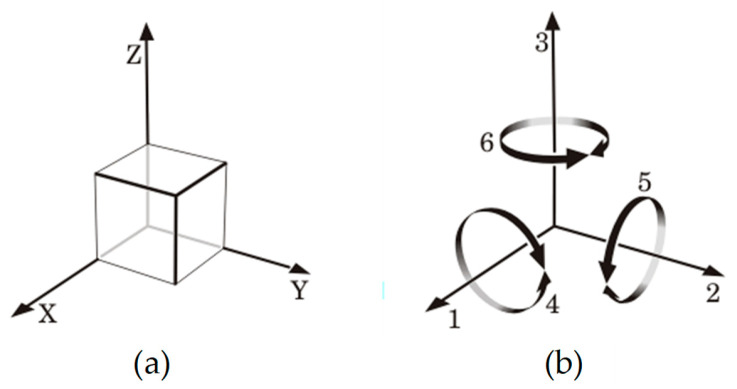
(**a**) Typical X-Y-Z orthogonal system. (**b**) Orthogonal system adapted to the analysis of the piezoelectric effect.

Let us assume a piezoelectric material in the typical X-Y-Z orthogonal system shown in Figure 17a, where the Z-axis is the direction of the electrical field established during the manufacturing process in the case of a synthetic material. The axes X, Y, and Z in Figure 17a are usually represented as the 1, 2, and 3 directions, respectively, as shown in Figure 17b. Additionally, the orthogonal system in Figure 17b also includes the rotational axes 4, 5, and 6, which identify the shear in axes 1, 2, and 3, respectively [32]. In Figure 17b, the polarization generated by the mechanical stress can appear in any of the three main directions (1, 2, and 3) of the piezoelectric material. In addition, such a polarization, in the flat operating region in Figure 16, is independent of the frequency of the mechanical signal.

Considering the previous assumptions and under conditions of a zero electric field (which will be practically obtained thanks to the virtual short-circuit of the charge amplifier explained in Section 4.4), the input–output characteristic of the direct piezoelectric effect can be modeled as [32,33]:

(23) $$\left(\begin{array}{c} \begin{array}{c} D_{1}\\ D_{2} \end{array}\\ D_{3} \end{array}\right)=\left(\begin{array}{cccccc} d_{11} & d_{12} & d_{13} & d_{14} & d_{15} & d_{16}\\ d_{21} & d_{22} & d_{23} & d_{24} & d_{25} & d_{26}\\ d_{31} & d_{32} & d_{33} & d_{34} & d_{35} & d_{36} \end{array}\right)\left(\begin{array}{c} \sigma_{1}\\ \sigma_{2}\\ \sigma_{3}\\ \sigma_{4}\\ \sigma_{5}\\ \sigma_{6} \end{array}\right)$$

where *D*_i_ (with *i* = 1, 2, and 3) is the electric displacement (in C/m^2^) obtained in the *i*-direction, *σ*_j_ (with *j* from 1 to 6) is the mechanical stress (in N/m^2^) applied in the *j*-direction following Figure 17b, and *d*_ij_ is the piezoelectric charge coefficient (quantified in C/N) that relates the charge density developed in the *i*-direction (under short-circuit conditions) when the stress is applied in the *j*-direction. For example, *d*_31_ relates the charge density developed in direction 3 when the stress is applied in direction 1, whereas *d*_15_ relates the charge density developed in direction 1 when the stress is applied in direction 5 (i.e., shear stress in direction 2). Note that the electric displacement quantifies the charge density displaced in the *i*-direction as a consequence of the mechanical stress applied in the *j*-direction. These displaced charges will induce a polarization (with opposite polarity) of the electrodes placed in the *i*-direction [12]. Therefore, there is a direct correspondence between the electric displacement in (23) and the resulting polarization of the electrodes placed in the same direction.

Generally, only a few of the piezoelectric coefficients involved in (23) are different than zero. For example, the matrix of coefficients for quartz is [31]:

(24) $$\left(\begin{array}{cccccc} d_{11} & {-d}_{11} & 0 & d_{14} & 0 & 0\\ 0 & 0 & 0 & 0 & {-d}_{14} & {-2d}_{11}\\ 0 & 0 & 0 & 0 & 0 & 0 \end{array}\right)$$

with |*d*_11_| = 2.3 pC/N and |*d*_14_| = 0.7 pC/N; the sign of these two coefficients depends on the crystal cut and the standard being followed [33]. According to (24), the polarization of the electrodes in direction 1 can be caused by a mechanical stress in directions 1, 2, and/or 4; the polarization in direction 2 by stress in directions 5 and/or 6; and no polarization appears in direction 3. On the other hand, for PZT, the corresponding matrix is:

(25) $$\left(\begin{array}{cccccc} 0 & 0 & 0 & 0 & d_{15} & 0\\ 0 & 0 & 0 & d_{15} & 0 & 0\\ d_{31} & d_{31} & d_{33} & 0 & 0 & 0 \end{array}\right)$$

The values of *d*_15_, *d*_31_, and *d*_33_ highly depend on the composition of the PZT. Coefficients *d*_31_ and *d*_33_ usually have opposite signs and they are at least an order of magnitude higher than the piezoelectric coefficients in quartz [34], thus resulting in a higher sensitivity. 

Let us consider that the piezoelectric material in Figure 17 is only subjected to a force *F*_1_ in direction 1. Then, assuming (24), we only have a polarization of the electrodes in direction 1 that is equal, under short-circuit conditions, to:

(26) $$D_{1}=d_{11}\sigma_{1}=d_{11}F_{1}/A,$$

where *A* is the sectional area of the material in direction 1. Assuming *D*_1_ = *Q*_1_/*A*, Equation (26) can be rewritten as follows:

(27) $$Q_{1}=d_{11}F_{1}$$

Therefore, in such a particular case, a charge *Q*_1_ proportional to *F*_1_ is generated between the electrodes placed in direction 1 of that material. 

Piezoelectric force sensors with a maximum measuring range of units, tens, hundreds, and even thousands of kN are commercially available. Considering a typical sensitivity of a few pC/N, the resulting output charge can be (at full scale) up to units, tens, hundreds, and thousands of nC, respectively.

### 4.4. Limitations

Mechanical piezoelectric sensors are not exempt from limitations, as occurs with other sensing technologies. Their main limitations are the following:

▪ Piezoelectric sensors are not valid for static measurements but are for dynamic measurements. The piezoelectric effect should be seen as a *dynamic process*—even if the material is kept compressed, the removed charges will not regenerate. New surface charges will appear either when further compressing or expanding the material. This explains the HPF behavior at low frequencies represented before in Figure 16, where the sensitivity tends to zero as the frequency decreases.
▪ The output of piezoelectric sensors has to be connected to a specific type of read-out circuit (so-called *charge amplifier*), otherwise both the HPF behavior and the sensitivity of the flat region in Figure 16 highly depend on the parasitic components of both the sensor and the interconnecting cable. Actually, some commercial piezoelectric sensors incorporate such a charge amplifier into the same module, so that they provide a voltage at the output instead of a charge. These, however, require some cables for the power supply of the charge amplifier. In such cases, the sensitivity is expressed in mV/N instead of pC/N.
▪ Piezoelectric sensors (especially those *manufactured* with synthetic materials) suffer from *thermal drifts*, as also indicated in Section 2.4 for semiconductor strain gauges. For example, the piezoelectric coefficients in (25) for PZT are temperature-dependent.

## 5. Comparison

In Table 1, a comparison of the main features of the three types of mechanical sensor explained before, including the subtypes, is carried out. The main advantage(s) of each type is highlighted in blue, whereas the main drawback(s) is shown in red. For strain gauges, the main advantage is the high sensitivity (especially, for the semiconductor type), but they suffer from thermal drifts. As for capacitive sensors, their main advantages are the low cost and low thermal drifts, whereas the main limitation is the complexity of the read-out circuit. Finally, piezoelectric sensors are a very good choice for high-bandwidth applications, although they do not offer a response in DC, and they are more expensive. As implied by Table 1, the ideal mechanical sensor does not exist, but each type offers pros and cons. The most appropriate mechanical sensor for a given application is the one that better adapts to the technical requirements of that application.

It is worth highlighting that, in recent years, extensive research has also been carried out on electronic interface circuits for mechanical sensors. For example, an interesting review about circuits for resistive sensors can be found in [35], whereas new amplifier circuits particularly designed for strain gauges were reported in [36]. As for capacitive sensors, an extensive review of read-out circuits was carried out in [37]. Specifically, a high-linearity front-end circuit for low-value GCS has been recently proposed in [38]. Finally, for piezoelectric sensors, modifications of the conventional charge amplifier have been lately suggested in [39,40]. In the former [39], the typical high-value feedback resistor is proposed to be replaced by a simple linear analog feedback network, whereas in [40], a novel method is suggested to compensate for the unwanted drift effect at the output.

## 6. Conclusions

In the 70th anniversary of the piezoresistive effect announced by C. S. Smith in 1954, a tutorial on mechanical sensors has been presented. For the three main types of mechanical sensor (i.e., strain gauges, capacitive sensors, and piezoelectric sensors), this tutorial has explained their operating principles, subtypes, input–output characteristics, and limitations, with the purpose of helping the reader to become familiar with and/or improve his/her knowledge about these sensors. In addition, the features of these three sensor technologies have been compared with each other, highlighting the main advantages and disadvantages.

## Figures and Tables

**Table 1:** Comparison between different types of mechanical sensor.

Feature	Strain Gauge		Capacitive Sensor		Piezoelectric Sensor	
	Metallic	Semic.	Single	Diff.	Natural	Synthetic
Measurement range	M	L	M		H	
Linearity	M	L	L ^(a)^	H ^(b)^	H	
Sensitivity	**L**	**H**	L	M	L	M
Thermal drifts	M	**H**	**L**		M	H
Read-out circuit complexity	M	L	**H**		M	
Bandwidth	M		M		**H**	
DC response	**Yes**		**Yes**		**No**	
Mechanical robustness	M		M		H	
MEMS compatibility	Yes		Yes		Yes	
Cost	M		**L**		**H**	

Abbreviations: L: Low; M: Medium; H: High. ^(a)^ Specially for SCS exposed to distance variations. ^(b)^ Assuming compensation of the electrostatic forces in the case of a capacitive MEMS.

## Data Availability

The data that support the findings of this study are available upon reasonable request from the author.
